# Mitochondrial DNA sequencing reveals association of variants and haplogroup M33a2′3 with High altitude pulmonary edema susceptibility in Indian male lowlanders

**DOI:** 10.1038/s41598-019-47500-1

**Published:** 2019-07-29

**Authors:** Swati Sharma, Sayar Singh, Rajinder K. Gupta, Lilly Ganju, Shashi Bala Singh, Bhuvnesh Kumar, Yamini Singh

**Affiliations:** 1 Defence Institute of Physiology and Allied Sciences, Timarpur, Delhi, 110054 India; 2 NIPER, Hyderabad, Balanagar, Hyderabad, 500037 India

**Keywords:** Genetic association study, Mitochondrial genome

## Abstract

High Altitude Pulmonary Edema (HAPE) is a threatening disorder caused due to acute exposure to high altitude above 3000 m. Apart from multiple factors involved, the genetic factors also play an important function in the pathogenesis of HAPE. This study aims to evaluate the role of mtDNA polymorphism and their association with haplogroup in understanding the etiology of HAPE. In this study, all the HAPE susceptible and acclimatized control subjects could be classified into nine haplogroups pertaining mostly to Macrohaplogroup M and U. The frequency of haplogroup M was significantly higher in HAPE susceptibles whereas the haplogroup M33a2′3 was found only in HAPE susceptibles. The variant G4491A and A4944G of MT-ND2, A14002G of MT-ND5, and C8562T of MT-ATP8, were definition site of haplogroup M33a2′3. The frequency of A10398G of MT-ND3, A8701G of MT-ATP6 and C14766T of MT-CYB genes were significantly higher in HAPE susceptibles. mtDNA copy number also plays a significant synergistic role in HAPE susceptibility. Our findings suggests that variants in MT-ND2 and MT-ND5 were predicted to confer decreased protein stability in HAPE susceptibles and in particular, highly conserved variants G4491A, A4944G and A14002G associated with haplogroup M33a2′3 may be the primary cause of susceptibility to HAPE in Indian male lowlanders.

## Introduction

Mitochondria are semiautonomous cytoplasmic organelles of the eukaryotic system that imparts essential functions in energy metabolism, free radical production, calcium homeostasis and apoptosis^1–3^. In humans, mitochondrial DNA (mtDNA) is the extranuclear circular molecule that spans 16,569 bp and encodes genes of 13 proteins, 2 rRNAs and 22 tRNAs, as well as 121 bp regulatory region (the D- Loop), all of which are components of the electron transport chain (ETC), and are essential for oxidative phosphorylation (OXPHOS)^4^. The OXPHOS system has a mixed genetic origin, nuclear and mitochondrial. It consists of approximately 90 different structural protein subunits of which thirteen are encoded by the mtDNA in humans^5^. The mitochondrial genome encode seven subunits of complex I, one subunit of complex III, three subunits of complex IV and two subunits of complex V^6^.

High altitude Pulmonary Edema (HAPE) is a life threatening, multifactorial complex disease condition which occurs due to rapid ascent to high altitude ~ 3000 m above sea level, where the oxygen content in air is only 60% of that at sea level^7^. Genetic predisposition to the disease is largely unknown, but it would be a function of gene environment interaction^8^. The role of genetics in determining the individual’s susceptibility to HAPE is unclear. Variants of some genes CYP11B2, ACE, EDN1(nuclear genes) were previously investigated for their association with HAPE^9^. The pathophysiology of HAPE is attributed to several etiological factors like pulmonary hypertension, hypoxic pulmonary vasoconstriction, injury to the blood-gas barrier, elevated capillary pressure and increased pulmonary vascular permeability^10^. During severe hypobaric hypoxia, there is an increased generation of reactive oxygen species (ROS) and reactive nitrogen species (RNS), causing mitochondrial dysfunction due to oxidative stress, consequently resulting in reduced ATP production^11^. It has been also reported that mitochondrial ROS mutate mtDNA leading to accumulation of somatic mitochondrial DNA mutations which may deplete the energy source^12^. Mitochondria are critical for hypoxic adaptation and acclimatization at high altitude by undergoing several mechanisms of adaptation in order to meet the cellular energy demand^13^. Thus, mitochondria may play an important role in high altitude adaptation/acclimatization, and the polymorphism in mtDNA might lead to susceptibility to HAPE.

The mtDNA differs from nuclear genome in number of characteristics features, including frequent mutation, maternal inheritance, high copy number, and lack of recombination. All these features offer the potential for investigation of human evolution and origin^14^. Human ancestors were originated from Africa about 100,000–200,000 years ago and spread worldwide via different routes according to the ‘Out of Africa’ model^15^. Mitochondrial DNA haplogroups are characteristic cluster of tightly linked mitochondrial DNA (mtDNA) polymorphism that forms continent-specific genotypes^16^. Studies also showed that certain mitochondrial haplogroups are predisposed to high-altitude diseases while others can protect against mountain sickness^17,18^. Research on mitochondrial genome shows that haplotype 3010G–3970C is significantly different in frequency between high and low altitude populations suggesting that this haplotype may be responsible for adaptation to low-oxygen environment^19^. Mitochondrial haplogroup N9b is protective against myocardial infarction in Japanese men^20^. Mitochondrial disorders are a group of clinically heterogeneous diseases, commonly defined by a lack of cellular energy due to oxidative phosphorylation defects. The change in mitochondrial function is important in understanding the mechanism for body’s acclimatization to hypoxia, and it is related to modifications of mtDNA sequences. These modifications can include polymorphisms, content changes, and sequence deletion^21^. In this study, we hypothesized that mtDNA haplogroup and polymorphism maybe an important factor in the susceptibility of HAPE. To confirm this hypothesis whole mitochondrial genome sequencing of HAPE susceptible and acclimatized control individuals of Indian population has been done. The secondary structural prediction was also carried out for identifying the conformational changes in nonsynonymous variants associated with the susceptibility to HAPE.

## Results

### Data summary of sequencing and quality control

**Figure 1:**
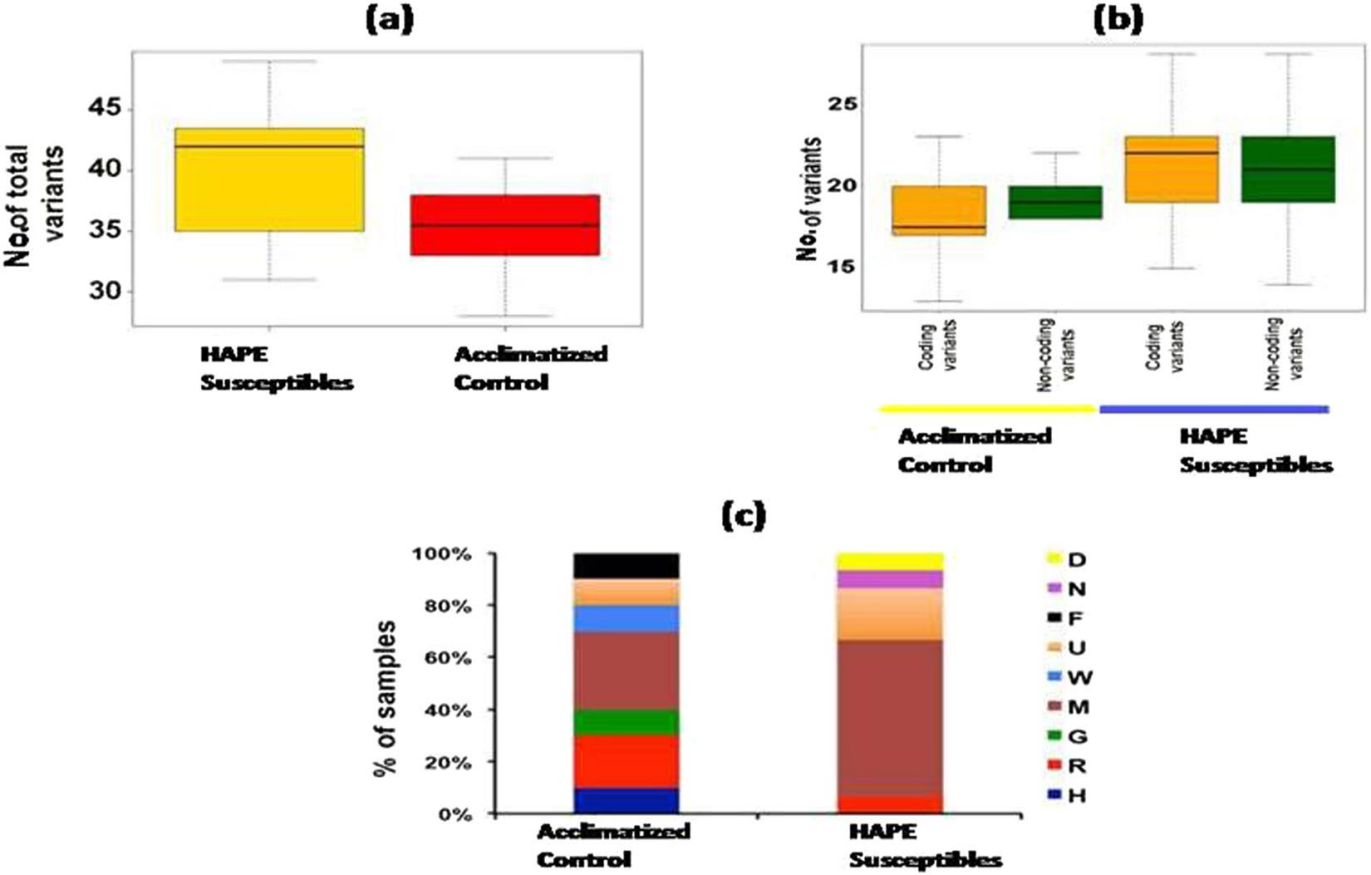
Distribution of variants and haplogroups in HAPE susceptibles and acclimatized control. (**a**) Total numbers of variants were higher in HAPE susceptibles compared to Acclimatized control (**b**)Total number of coding and noncoding variants were higher in HAPE susceptibles (**c**) Shows percentage of haplogroup in HAPE susceptibles and Acclimatized control. Frequency of haplogroup M is higher in HAPE susceptibles.

Mitochondrial DNA of 35 human blood samples was isolated and was sequenced using Illumina Hi Seq platform to investigate the base changes in diseased and acclimatized control. Close to 90% of total data generated was over Q > 30 Phred score. On an average 97% processed data was aligned to the reference human genome and among the align reads 70% were aligned to mitochondrial genome with mapping quality ≥ Q29 and insert size ≥ 100 bp; an average of 17% of the passed reads were found to be duplicates. After removing duplicates, an average depth of 4600X was observed. For all the samples, 100% of the mitochondrial genome region was covered. Total number of variants detected in HAPE susceptible group was higher as compared to acclimatized control group (Fig. 1a). On an average we have found 42 variants per sample in HAPE susceptible group in mitochondrial genome. The coding and non coding variants were observed to be higher in the HAPE susceptible group (Fig. 1b).

### Mitochondrial haplogroups distribution among HAPE susceptible and acclimatized control

After sequencing of mtDNA, haplogroup distribution was analyzed in the two groups. The results show difference in haplogroup distribution between HAPE susceptible and acclimatized control groups. A total of nine haplogroups were observed among the two groups. The mitochondrial haplogroup among non acclimatized (HAPE susceptible) and acclimatized control belong mostly to M and U haplogroups. Haplogroups F, W, G, and H were found to be negatively associated with HAPE susceptible. The frequencies of haplogroup M in the HAPE susceptible was significantly higher compared with those of the acclimatized control (Table 1). 60% of the HAPE susceptible samples belong to M haplogroup, which is a major haplogroup of South Asia. The proportion of U haplogroup was slightly higher in HAPE susceptible (Fig. 1c).

**Table 1:** Distribution of the haplogroup frequencies between HAPE Susceptibles and Acclimatized control along with their p valuewhich is calculated from Fischer exact test.

Haplogroup	Acclimatized Control (n = 20) (%)	HAPE Susceptibles (n = 15) (%)	p value
D	0	6.6	0.014
N	0	6.6	0.014
F	10	0	0.0015
U	10	20	0.07
W	10	0	0.0015
M	30	60	<0.00001
G	10	0	0.0015
R	20	6.6	0.0119
H	10	0	0.0015

Haplogroup M shows significantly higher frequency (60%) in HAPE susceptibles. *p value by Fisher exact test.

### Variant distribution in mitochondrial genome among HAPE susceptible and acclimatized control

**Figure 2:**
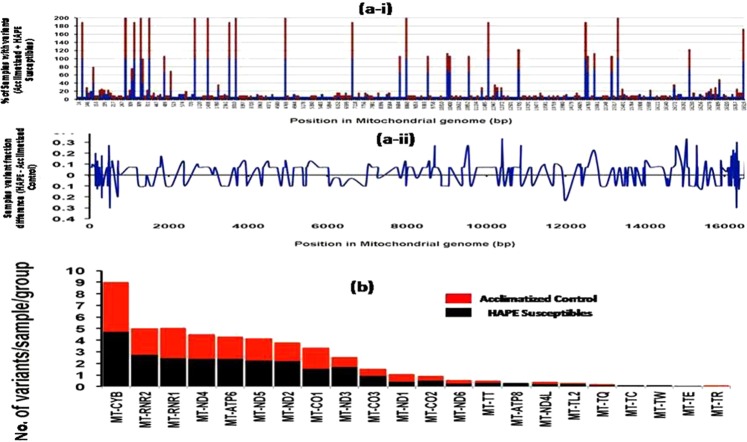
Gene distributions in mitochondrial genome. (**a-i**) Sample with variant presence in HAPE susceptibles and Acclimatized Control groups, Red bar represents percentage of HAPE susceptible samples where the Blue bar represents percentage of Acclimatized Control samples having variants at a given genomic coordinate, (**a-ii**) Difference fraction of variant percentage between HAPE susceptibles and Acclimatized Control sample. Positive value represent higher in HAPE susceptibles where negative value represents higher in Acclimatized Control (**b**) Showing total number of variants in each group wrt to mitochondrial genome.

From sequencing, overall 215 variants were found across all samples. Out of total we have found that 50% were unique to HAPE susceptible and 29% were unique to acclimatized control. Only 21% were found to be common between the two groups. The variants were compared at the genomic position level for the two groups. Figure 2a (i) shows the percentage of samples having variants at the genomic coordinate (X-axis of the graph). Figure 2a (ii) shows the difference in variant concentration across mitochondrial genome which indicates that a large number of variants were at the start or the end of the mitochondrial genome. The total number of variants found in each sample of each group with respect to number of genes is shown in Fig. 2b. Total number of unique variants was found to be higher in the HAPE susceptibles.

### Association of mtDNA variants with HAPE susceptibility

In order to find an association of HAPE with mitochondria, the variants obtained from mitochondrial DNA sequencing were analyzed in between two groups and it was observed that the variants G4491A, A4944G, C8562T, and A14002G were found only in HAPE susceptibles with p < 0.05 (Table 2). The frequency of A8701G, A10398G, C14766T variants in HAPE susceptible were significantly higher compared with acclimatized control (p < 0.05). The conservation analysis of seven variants (A14002G, A4944G, C14766T, A8701G, G4491A, C8562T, A10398G) that may cause amino acid changes showed that A14002G, A4944G, C14766T, A8701G were highly conserved (CI = 75%, 73%, 69%, 67%, respectively) whereas A4491G, C8562T, A10398G were not conserved during evolution (Table 3).

**Table 2:** Variant frequency distribution of alleles along with their respective genes and amino acid changes of HAPE susceptiblesand acclimatized control.

Allele	Location	Amino acid Change	HAPE Susceptibles Frequency (%) (n = 15)	Acclimatized Control Frequency (%) (n = 20)	p value
G4491A	MT-ND2	Val8Ileu	13.3	0	0.0002
A4944G	MT-ND2	Ileu159Val	13.3	0	0.0002
C8562T	MT-ATP8	Pro66Leu	13.3	0	0.0002
A14002G	MT-ND5	Thr556Ala	13.3	0	0.0002
A8701G	MT-ATP6	Thr59Ala	66.6	20	0.0132
A10398G	MT-ND3	Thr114Ala	73.3	20	0.0024
C14766T	MT- CYB	Thr7Ile	100	45	0.0005

Values are presented as frequency in percentage and the p value is predicted by Fischer exact test (p < 0.05).

**Table 3:** Conservation analysis of SNP between HAPE susceptibles and acclimatized control presented as percentage of highlyconserved amino acid during evolution.

Allele	Conservation index (%)	Reported (population context)
G4491A	17%	Yes
A4944G	73%	Yes
C8562T	30%	Yes
A14002G	75%	Yes
A8701G	67%	Yes
A10398G	36%	Yes
C14766T	69%	Yes

A14002G is 75%, A4944G is 73%, A8701G is 67% and C14766T is 69% conserved during evolution.

### Association of mtDNA variants with haplogroup in HAPE susceptible

The variants G4491A, A4944G, C8562T, and A14002G are definition site of haplogroup M33a2′3. In this study we observed that the haplogroup M33a2′3 with higher frequency of 13.3% was found only in HAPE susceptibles (p = 0.0002) (Table 4), whereas haplogroup M33a2′3 was not found in acclimatized control because the mutations G4491A, A4944G, C8562T, and A14002G were not observed in acclimatized control. Variants A8701G, C14766T and A10398G were observed in higher frequency in HAPE susceptibles.

**Table 4:** Macrohaplogroup M and frequency of subhaplogroups in HAPE Susceptibles and acclimatized control. M33a2′3 shows13.3% frequency in HAPE Susceptibles (p < 0.05).

Macrohaplogroup	Subhaplogroup	HAPE Susceptibles (N = 15) %	Acclimatized Control (N = 20) %	p value
M	D4i	6.6	0	0.014
	M65b	6.6	0	0.014
	M33a2′3	13.3	0	0.0002
	M2a1	6.6	0	0.014
	M39a1	6.6	0	0.014
	M35b4	6.6	0	0.014
	M36d	6.6	0	0.014
	M3a1	6.6	0	0.014

*p < 0.05 by Fischer exact test.

### Genotypic analysis of mtDNA variants

Total number of polymorphic sites in MT-ND2, MT-ND5, MT-ND3, MT-CYB, MT-ATP6 and MT-ATP8 genes in all the subjects is given in Table 5. The polymorphic sites G4491A, A4944G of MT-ND2, A10398G of MT-ND3, A14002G of MT-ND5, C14766T of MT-CYB, A8701G of MT-ATP6, and C8562T of MT-ATP8, genes were genotyped for their association with haplogroup. Interestingly we have found through genotyping that the frequency of all the seven variants were significantly higher and G4491A, A4494G, A10398G and A14002G were associated with haplogroup M33a2′3. We have also observed that there was a significant increase in mtDNA copy number in HAPE susceptibles compared to acclimatized control.

**Table 5:** Gene Diversity parameters showing polymorphic sites, haplotype, average number of differences and nucleotides in therespective mitochondrial genes in all subjects.

Gene sites	No. of haplotype	No. of diversity	Average no. of differences	Nt Polymorphic
MT-ATP6	15	14	0.903	0.00261
MT-ATP8	4	4	0.297	0.0019
MT-CYB	23	17	0.953	0.00301
MT-ND2	22	14	0.813	0.00183
MT-ND3	7	6	0.66	0.00414
MT-ND5	33	19	0.96	0.00188

MT-ND5, MT-ND2 and MT-CYB showing higher number of polymorphic sites.

### Secondary structure prediction and analysis of NADH dehydrogenase subunits II and V

**Figure 3:**
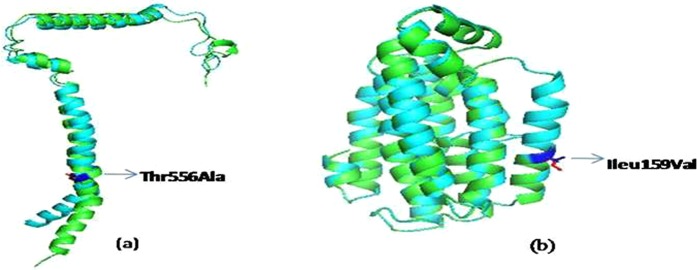
Overlapped secondary structure of Complex I subunit. (**a**) Showing overlapped secondary structure of MT-ND5 gene of HAPE susceptible and Acclimatized Control. The dark blue portion suggests a change in amino acid from Threonine to Alanine at position 556. (**b**) Overlapping of secondary structure of MT-ND2 gene in HAPE susceptible and acclimatized control showing a change from Ileu159Val. Changes in amino acids and negative free energy change (∆∆G) shows destability of protein in HAPE susceptibles.

To analyze the effect of mitochondrial genetic variations on encoded protein, we have further studied the secondary structure of mitochondrial NADH dehydrogenase subunits II and V of Complex I of OXPHOS system for investigating the structural variations cause by specific base substitution. The variant A14002G of MT-ND5 gene has change in amino acid at Thr556Ala in HAPE susceptibles. On superimposition of secondary structure of HAPE susceptible and acclimatized control, a change of amino acid Threonine to Alanine was observed as shown in Fig. 3a. The free energy change (∆∆G) of this mutation was −0.71. Similarly, in MT-ND2 gene, the variant A4944G was found in amino acid Ileu159Val in HAPE susceptible. On comparing the secondary structure of HAPE susceptible and acclimatized control it has been observed that the mutation of amino acid Isoleucine to Valine was present in HAPE susceptible (Fig. 3b). Also, the free energy change of this mutation in HAPE susceptible is less than zero i.e. −0.09.

## Discussion

Mitochondria are major ATP production site in mammalian cells. The mtDNA polymorphism affects the function of mitochondria^22^. High Altitude Pulmonary Edema caused under hypoxic condition at high altitude also have major impact in the energy generating OXPHOS system of mitochondria.

In this study, we compared the whole mtDNA sequences of acclimatized control and HAPE susceptibles. We have found that frequencies of the A8701G, A10398 and C14766T variants in HAPE susceptibles were significantly higher compared to acclimatized control (p < 0.05) and the variants G4491A, A4944G, C8562T, and A14002G were found only in HAPE susceptibles. Furthermore, the frequencies of haplogroups F, W, G, and H are higher in acclimatized control. In addition, the frequency of M in HAPE susceptibles was significantly higher as compared to acclimatized control. The subhaplogroup M33a2′3 was specifically found in HAPE susceptibles. Studies on mtDNA variation in India^23,24^ have reported the presence of macrohaplogroup M in a majority of the individuals (~60%). The frequency of M was observed to be 58% among the caste and 72% among the tribal populations^25–28^. Among the defined haplogroups of M: M2, M3, M4, M5, M6, M18, M25, M30, M31, M32, M33, M34, M35, M36, M37, M38, M39, M40, M41, M48, M49 and M50 are the India-specific lineages^29^ In previous studies, it has been reported that Haplogroup M33 is defined by the coding region substitution at np 2361. One of the studies suggests that haplogroup M33 in seven tribes have sub-branches M33a, M33b, M33c. M33a has been further subdivided into three new subhaplogroups i.e, M33a1, M33a2, M33a3. Lepcha tribes from Sikkim, have been grouped under M33a1a, whereas Dongri Bhill tribe from West India (Madhya Pradesh, Rajasthan) have been categorized under M33a1b^30^. M33a2 haplogroup has been assigned to the tribes of Maharashtra. Sun *et al*.^31^ reported this haplogroup in Rajbansi population of West Bengal and Brahmins of Uttar Pradesh. A sub-haplogroup of M33, M33a (8562–15908), was reported in Tadvi population of Gujarat and among Khasi Khumic populations (~5%), with an exceptionally high frequency (~55%) among the Garo of Meghalaya^32^. The TMRCA of haplogroup M33 was reported to be ~50, 000 ybp^33,34^. In our study we suggests that coding region substitution in mtDNA G4491A, A4944G, C8562T and A14002G variants were associated with subhaplogroup M33a2′3 in HAPE susceptibles in Indian male lowlanders. M33a2′3 mtDNAs themselves increase in frequency with HAPE susceptibles. M33a2′3 were only found in HAPE susceptibles, therefore all four variants (G4491A, A4944G A14002G and C8562T) and haplogroup M33a2′3 mtDNAs are enriched in HAPE susceptibles. Variants in this haplogroup may lead to change in mitochondrial function enabling susceptibility to the hypoxic environment. Therefore we speculate that M33a2′3 subhaplogroup might be susceptible to HAPE in Indian male lowlanders. In the present study, the subjects were Indian Army recruits, who deployed at high altitude following acclimatization schedule that reduce the risk of HAPE. Therefore the family history could not be taken and the sample size was limited. Further genetic lineage studies need to be investigated to understand the development of HAPE and its association to mtDNA on a large sample size.

NADH dehydrogenase is a part of the Complex I of OXPHOS system. Each subunit of NADH dehydrogenase has some assigned function. ND2, ND4 and ND5 were suggested to be the actual proton pumping device because of their sequence homology with the class of Na+/H+ antiporters^35^. Rute R suggests that missense mutations that cause amino acid changes in subunits of NADH may interfere with the efficiency of proton pumping process by disrupting/improving the long range redox link conformational changes that could hinder or improve the proton translocation and thus the functionality of NADH dehydrogenase^36^. Another study on mitogenomics suggests that positive selection in mitochondrial NADH dehydrogenase subunits ND2, ND3, ND4, and ND5 drives to better adapt to energy requirement to high altitude of Tibetan Plateau^37^. In our study we have also revealed that mutations, G4491A, A4944G occur in NADH dehydrogenase subunit 2, A10398G of NADH dehydrogenase subunit3 and A14002G in NADH dehydrogenase subunit 5 of mtDNA specifically characteristic to haplogroup M33a2′3 cause amino acid changes. Earlier studies suggested that amino acid replacements resulting in a dissimilar amino acid are generally more deleterious than replacements resulting in an amino acid with similar properties^38^. The pathogenic SNP in mtDNA may be associated with susceptibility to complex diseases^39^. The effect is most likely due to change in the amino acid sequence of protein coding genes. A study on structural simulation and function analysis on ND2(V81I) and COX(V38I) shows that replacement of amino acid cause change in the protein conformation and protein stability, suggesting an adaptive variation^40^. Studies on human glioma cell lines demonstrated that a mutation T14634C in mtDNA encoded Complex I ND6 subunit (NADH dehydrogenase) causing amino acid change M14V significantly disrupt the orientation of entire ND6 protein within mitochondrial membrane and would alter the interaction of the individual helices of each protein thus associated with hypoxia sensitive phenotype^41^. The mtDNA variation G4491A causes changes in amino acid from Val8Ileu and A4944G causes changes in amino acid from Ileu159Val, A10398G causes changes from Thr114Ala and A14002G with amino acid change Thr556Ala, could be considered as potentially functional polymorphism. The replacement of amino acid may cause change in the hydrophobicity/ hydrophilicity, charges and conformation that may affect the secondary, tertiary or quaternary structure of proteins and its function^42^. Val, Ileu, Ala is hydrophobic uncharged amino acid whereas Thr is a hydrophilic polar amino acid. The negative value of free energy change (G) by STRUM confers that the nonsynonymous mutations suggests destabilization of protein NADH dehydrogenase of Complex I, that functions in the transfer of electron from NADH to the respiratory chain, thus may influence the function of NADH dehydrogenase and OXPHOS system in mitochondria.

The MTCYB gene is a component of the ubiquinol-cytochrome c reductase complex (complex III or cytochrome b-c1 complex) that is part of the mitochondrial respiratory chain. The b-c1 complex mediates electron transfer from ubiquinol to cytochrome c, contributes to the generation of a proton gradient across the mitochondrial membrane that is then used for ATP synthesis. A significant nonsynonymous mutation of MT-CYB gene, C14766T, is highly conserved and found in HAPE susceptibles as well as in acclimatized control individuals. The sift score for pathogenicity was found of be 0.03, suggests that the mutation is highly deleterious. However the frequency of this mutation in HAPE susceptibles was relatively higher (100%) than acclimatized control(45%). The missense mutation causing amino acid change Thr7Ileu in MT-CYB protein might be associated with the pathogenicity in HAPE susceptibles. This mtDNA C14766T polymorphism result in Threonine to Isoleucine change at amino acid residue 7 in Cytochrome b (CYB), both carries no charge. Threonine is hydrophilic acid with hydroxyl group and is involved in protein interaction after phosphorylation while Isoleucine is nonpolar, uncharged, low molecular weight and is hydrophobic^43^. It may be hypothesized that this variant may be associated with high altitude sickness.

The variant A8701G occurs in subunit 6 and variant C8562T in subunit 8 of mitochondrial ATP synthase Complex V, which is responsible for last step of oxidative phosphorylation and enables protons to flow back to the matrix and uses the released energy to synthesize ATP. It is composed of at least 16 subunits, of which 2 (ATP6 and ATP8) are encoded by the mtDNA. These variants causes change in amino acid from Thr59Ala and Pro66Leu respectively. A previous study illustrated that mutations of the *ATP6* gene were associated with a deficiency in the stability of complex V^44^. The nucleotide change A to G at position 8701 and C to T at position 8562 in *ATP6* gene, part of the ATP synthase protein, has been demonstrated to reduce ATP synthesis and significantly impair the assembly or stability of the ATP synthase^45^. Previous study showed that by using transmitochondrial hybrid cells, mtSNPA8701G cause abnormal results in impaired mitochondrial pH and intracellular calcium dynamics and is suspected to be associated with the pathogenesis of some diseases^46^. Another study also suggests that mtDNA mutations in the protein subunits of OXPHOS cause biochemical deficiency in the Complexes^47^. Mcfarland R demonstrate that mutations in ND3 gene T10191C and T10158C cause disproportionatly greater reduction in the enzyme activity of Complex I^48^. A study on myopathic syndrome demonstrate that 5591G > A transition in mitochondria tRNA^ala^ gene segregates with cytc oxidase deficiency in muscle fibres^49^. No previous study has been done till now to associate mtDNA polymorphism with OXPHOS dysfunction in HAPE. Therefore it may be suggested that multiple types of mutations in mtDNA causes dysfunction of oxidative phosphorylation (OXPHOS) in HAPE susceptibles.

The conservation analysis showed that the variants G4944A (Ileu159Val), A14002G (Thr556Ala), C14766T (Thr7Ileu) and A8701G(Thr59Ala) are in the evolutionary conserved region of MT-ND2, MT-ND5, MT-CYB, and MT-ATP6 respectively and changes in polarity of amino acids may change the predicted secondary structure & function of respective genes, thus any alteration is highly harmful leading to susceptibility to HAPE. mtDNA copy number is an important factor in regulating human bioenergy process such as balance between ATP production and thermogenesis^50^. Consequently, variation in mtDNA content between individuals could contribute to various physiological trait and diseases^51,52^. It has been shown that increase in mtDNA content is associated with decline in environmental temperature and mtDNA may contribute to the human adaptation to different environment^53^. A study on mtDNA copy number suggests that increased mtDNA copy number in MSS CRC (colorectal cancer) significantly promoting progression by upregulating OXPHOS function^54^. Established genetic and biochemical studies strongly suggests that transcription factors acting together can bring about mitochondrial biogenesis and increase OXPHOS^55^. Our study showed increase mtDNA copy number that may affect the OXPHOS. Thus, previous studies supports our present study that higher mtDNA copy number may affect the expression level of energy metabolism enzymes involves in ATP synthesis, though expression studies are required to validate these results.

The transfer of electrons during oxidative phosphorylation results in the generation of reactive oxygen species (ROS), namely superoxide, at low levels^56^. In response to hypoxia, HIF transcription activity and mitochondrial function indirectly regulates each other^57^. Earlier studies suggested that acute hypoxia leads to increased mitochondrial generation of reactive oxygen species (ROS) that prevents the hydroxylation of HIF1 alpha and increase HIF 1 alpha transcription of genes (LDHA, PDK1, BNIP3, COX4-2, miroRNA) that reduce mitochondrial respiration and ROS production^58^. It has been also reported earlier that baseline elevation of HIF1 alpha is associated with HAPE susceptibility^59^. It may be suggested from this study that mutations in mitochondrial genes of Complex I, III and V that cause dysfunction of OXPHOS may enhance HIF transcriptional activities of genes that encode transcription factor, which is regulated by inactive HIF-1 alpha hydroxylases under hypoxic conditions.

In summary using HAPE susceptibles and acclimatized control, we have analyzed 35 human individuals for association of mtDNA haplogroup with individual risk of HAPE. Our findings reveals that mtDNA haplogroup M33a2′3 may be the risk factor for HAPE in Indian lowlanders. Haplogroup M33a2′3 associated with mutations G4491A, A4944G of MT-ND2 gene, A14002G ofMT-ND5 gene and A8562T of MT-ATP-8 gene was overrepresented in the HAPE susceptibles that may cause reduction in the activity of complexes of mitochondrial respiratory chain in OXPHOS system, hence these mutations could imply the possibility of selection of susceptibility and mitochondrial dysfunction in HAPE susceptibles.

## Material and Methods

### Population sampling and DNA extraction

A total of n = 35 unrelated, age matched, lowlanders male individuals of Indian origin, out of which healthy control sojourners (n = 20), who did not developed HAPE on induction to high altitude at Leh, situated at 3250 m above sea level, and maladapted sea level resident of Indian origin (n = 15) who developed at least one episode of HAPE after air induction to Leh, were recruited for the study. HAPE was diagnosed radiographically. The individuals who do not develop HAPE, stayed at high altitude (3250 m) for tenure of 2 years after following the acclimatization schedule were considered as acclimatized control. All individuals have not been previously lived at high altitude (>3000 m) at some point of their lives. All the individuals of both the groups followed acclimatization schedule for the initial six days of induction to high altitude which involve complete rest for first two days followed by graded increase in physical activity. All individuals were checked for medical history and were non smokers, & non-obese. Within last six months, none of the participants has resided above 2000 m before baseline measurements. 3–4 ml Blood samples were collected in 6 ml K2 EDTA tubes from all the subjects. Genomic DNA was extracted from whole blood using QIAmp DNA blood kit according to the recommendations of the manufacturer. All the experimental protocols were approved by Defence Institute of Physiology and Allied Sciences (DIPAS), Delhi ethics committee for scientific experiments. Individuals were asked to participate in the study by signing a written informed consent, as approved by the Institutional Ethical Committee of Defence Institute of Physiology and Allied Sciences as per Ethical Guidelines for Biomedical Research on Human Participants of Indian Council of Medical Research. All the Subjects were above 18 years old. All methods were carried out in accordance with the approved guidelines and regulations.

### mtDNA amplification and sequencing

The entire mitochondria genome is amplified in two fragments from the whole cell DNA extract using specific primers. The fragments are overlapping and have sizes of ~8338 and 8647 basepairs. The primer pair used were L644 (GACGGGCTCACATCACCCCATAA) and H8982 (GCGTACGGCCAGGGCTATTGGT) for fragment 1 and L8789 (GCCACAACTAACCTCCTCGGACTCCT) and H877 (GGTGGCTGGCACGAAATTGACC) for fragment 2^60^. Long Range PCR was done using Expand Long Range dNTPack (Sigma Aldirch cat no. 4829034001). The reaction mixture of volume 25 ul was made with 5 ul, 5X expanded Long range buffer, 1.25 ul (500 uM) dNTP, 10 uM each primer, 3%DMSO, 0.5 ul 2.5 U Expand long range enzyme mix, 100 ng DNA for each fragment. The cycling conditions were as follows: One cycle at 92 °C for 2 min for an initial denaturation followed by 10 cycles of denaturation at 92 °C for 10 s, primer annealing at 66 °C for 15 s, primer extension at 68 °C for 8 min 30 s for fragment 1 while for fragment 2, 15 cycles of the same denaturation, annealing was done & the final extension at 68 °C for 7 min for both fragment 1 and fragment 2. The PCR product is run on 0.8% agarose gel, and the appropriate band is extracted from the same using Invitrogen PureLink Quick Gel Extraction and PCR Reaction Cleanup Kit (Catalog Number - K220001). The two amplicons from each sample are pooled in equal concentrations and the pool is taken for library preparation using Illumina TruSeq Nano DNA Library Preparation Kit (Catalog Number - FC-121-4001), following the manufacturer’s protocol without any modifications. The libraries are sequenced on Illumina’sHiSeq 2500 instrument (0.5 GB per sample).

### Sequence data analysis

Based on the quality report of fastq files sequence reads were trimmed to only high quality sequence for further analysis. The paired end reads were aligned to the reference human genome Feb 2009 release downloaded from UCSC database (GRCh37/hg19). We have used Revised Cambridge reference sequence (rCRS) as mitochondrial reference genome (http://www.mitomap.org/MITOMAP/Human/Mitoseq). GenomeAnalysisTKLite-2.3–9 toolkit Unified Genotyper (Broad institute, Cambridge, MA) was used to identify the single nucleotide variants (SNPs) and short Indels and was used to ensure quality variant calls. Sequences were aligned to hg19 using BWA program (v.0.7.8). The conservation index of the variants was analyzed using Mitotool (www.mitotool.org). Variant annotation is performed using Medgenome’s annotation tool and VeP program (ENSEMBL75). Haplotype diversity, average number of differences (K), and nucleotide diversity (п) of the genes were determined using DnaSP V5 software.

### mtDNA haplogroup classification and phylogenetic analysis

Haplogroup along with the confidence score for each sample haplotype is predicted using Haplogrep program under default setting according to the criteria of MITOMAP. Series of affecters were considered when appraising mtDNA variants of the particular branches of each haplogroup, the calescence time of variation distribution and the location of protein or RNA-encoded gene based substitution. All these factors were included in phylogenetic analysis and construction of Haplogroup. For the phylogenetic analysis three samples were taken through NCBI (accession ID: AF346972.1, EF060346.1, AF346987.1), each one from Chinese (M9a1), Ethopia (M1a4) and African (L1c1d) ancestry. Multiple sequence alignment was performed using ClustalW program and NJ tree was generated using 1000 bootstrap option (Mutliple_seq_alignment.aln).

### mtDNA variant genotyping

Amplification of DNA fragments for sequencing was performed using specific primers of genes MT-ND2, MT-ND5, MT-ATP6, MT-ATP8, MT-CYB to genotype the variants (G4491A, A4944G, C8562T, A14002G, A8701G, C14766T and A10398G) polymorphism. The primers were designed using software NCBI-Primer-BLAST and were synthesized by Chromous Biotech. The samples were run on a DNA Analyzer (ABI sequencer 3130 genetic analyzer, assembled and were compared to the rCRS reference sequence. The primer sequences and reaction conditions were described in Table 6. Haplogroup and variant analysis validation has been performed using Mitotool (www.mitotool.org).

**Table 6:** Specific PCR Primer sequence and reaction conditions of MT-ND2 (Product size ≈ 1000 bp), MT-ND5 (≈347 bp), MT-ATP6 (≈673 bp), MT-ATP8 (≈110 bp) and MT-CYB (≈523 bp).

Gene	Primers (bp)	Product	Annealing temp.	No. of cycles
MT-ND2	5′ATTAATCCCCTGGCCCAACC3′	1000	48	30
	5′TGGTAAGGGCGATGAGTGTG3′			
MT-ND5	5′CGGAAGCCTATTCGCAGGAT3′	347	48	28
	5′TGGAGGTGGAGATTTGGTGC3′			
MT-ATP6	5′CTCACCAAAGCCCATAAA3′	673	52	35
	5′AGGCGACAGCGATTTCTA3′			
MT-ATP8 5′ACTACCACCTACCTCCCTCAC3′		110	49	30
	5′GGATTGTGGGGGCAATGAATG3′			
MT-CYB	5′GGGACAGACCTAGTTCAATG3′	523	55	35
	5′CTGCGGCTAGGAGTCAATAA3′			

### Relative mtDNA copy number measurement

The relative mtDNA copy number was measured by the fluorescence-based quantitative real-time PCR by using DNA analysis kit for the determination of human mitochondrial DNA copy number, by the comparison of mitochondrial (mt) and nuclear (n) DNA measured by real-time PCR (Detroit R&D Cat No. MCN1). The primers for β actin were forward, 5′-CGGGAAATCGTGCGTGACAT-3′ and reverse, 5′-GAAGAAGGCTGGAAGAGTG-3′ whereas primers for mtDNA were forward, 5′-TACTCACCAGACGCCTCAACCG3′ and reverse, 5′-TTATCGGAATGGGAGGTGATTC-3′^56^. The PCR cycling conditions includes an activation step at 95 °C for 10 min, followed by 40 cycles for 15 sec at 95C, 1 min at 60 °C^61^, and is performed on a Real-Time PCR Detection system (BioRad, Hercules, CA). A positive control is included in the reaction. Each sample was run in triplicate for both mitochondrial gene and nuclear gene amplification.

### Structure prediction and analysis

The translated protein sequence of MT-ND2 and MT-ND5 genes were analyzed for secondary structure variations in HAPE and acclimatized individuals. Pfam domain analysis, structural template search was carried out using blast and multiple alignments. Secondary structure analysis was carried out using Raptor X property. The domain region of ND2 and ND5 was modeled using modeller standalone. The free energy change (∆∆G) was determined using STRUM tool which is a structure based prediction of protein stability changes upon single point mutation.

### Statistical analysis

Analysis of data was performed using SPSS software. Fischer exact test was used to assess the differences in haplogroup and SNP frequencies between the HAPE and acclimatized group. A two sided p value < 0.05 was considered to be statistically significant.
